# Metabolic engineering of *Moorella thermoacetica* for thermophilic bioconversion of gaseous substrates to a volatile chemical

**DOI:** 10.1186/s13568-021-01220-w

**Published:** 2021-04-23

**Authors:** Junya Kato, Kaisei Takemura, Setsu Kato, Tatsuya Fujii, Keisuke Wada, Yuki Iwasaki, Yoshiteru Aoi, Akinori Matsushika, Katsuji Murakami, Yutaka Nakashimada

**Affiliations:** 1 Graduate School of Integrated Sciences for Life, Hiroshima University, 1-3-1 Kagamiyama, Higashihiroshima, Hiroshima 739-8530 Japan; 2 National Institute of Advanced Industrial Science and Technology (AIST), 3-11-32 Kagamiyama, Higashihiroshima, Hiroshima 739-0046 Japan

**Keywords:** Gas fermentation, Metabolic engineering, Acetone production, Acetogen, Thermophile, *Moorella thermoacetica*

## Abstract

Gas fermentation is one of the promising bioprocesses to convert CO_2_ or syngas to important chemicals. Thermophilic gas fermentation of volatile chemicals has the potential for the development of consolidated bioprocesses that can simultaneously separate products during fermentation. This study reports the production of acetone from CO_2_ and H_2_, CO, or syngas by introducing the acetone production pathway using acetyl–coenzyme A (Ac-CoA) and acetate produced via the Wood–Ljungdahl pathway in *Moorella thermoacetica*. Reducing the carbon flux from Ac-CoA to acetate through genetic engineering successfully enhanced acetone productivity, which varied on the basis of the gas composition. The highest acetone productivity was obtained with CO–H_2_, while autotrophic growth collapsed with CO_2_–H_2_. By adding H_2_ to CO, the acetone productivity from the same amount of carbon source increased compared to CO gas only, and the maximum specific acetone production rate also increased from 0.04 to 0.09 g-acetone/g-dry cell/h. Our development of the engineered thermophilic acetogen *M. thermoacetica*, which grows at a temperature higher than the boiling point of acetone (58 °C), would pave the way for developing a consolidated process with simplified and cost-effective recovery via condensation following gas fermentation.

**Supplementary Information:**

The online version contains supplementary material available at 10.1186/s13568-021-01220-w.

## Introduction

Metabolic engineering of thermophilic microorganisms has several benefits compared to mesophilic microorganisms, such as a lower contamination risk, less energy required for cooling the fermentation system, and a faster production rate due to the advantageous thermodynamics (Sonnleitner and Fiechter 1983). One potential application is bioreactive distillation, which involves simultaneously fermenting volatile chemicals and collecting them by distillation (Zeldes et al. 2018). The bioreactive distillation has fewer steps and a lower cost of purification of target chemicals compared to the conventional fermentation processes. In addition, maintenance of the low concentration of fermentation products prevents the exposure of the chemicals to bacteria that inhibits bacterial growth and metabolism.

Acetone is a volatile chemical used as an industrial solvent and a precursor of important downstream products (Anbarasan et al. 2012; Peters et al. 2015). Currently, industrial acetone production depends on petrochemical phenol production via the cumene process. Although the cumene process is cost-effective and widely used, it has the risk of shortage of nonrenewable fossil feedstock. Therefore, there is a demand for an alternative process, and acetone production by bioconversion from renewable feedstock is one option.

Historically, acetone production by bioconversion has been studied as acetone-butanol-ethanol (ABE) fermentation. However, the focus is on alcohol production, and much effort is made for inhibiting acetone production as a by-product (Jiang et al. 2009; Luo et al. 2016; Xu et al. 2015). Metabolic engineering enables the development of acetone-producing strains such as *Escherichia coli* as the host (Bermejo et al. 1998). A few studies have reported thermophilic acetone fermentation from carbohydrates (Shaw et al. 2015; Straub et al. 2020). In addition, metabolic engineering of *Synechocystis* sp. PCC 6803, *Acetobacterium woodii*, and *Clostridium ljungdahlii* makes acetone production possible from CO_2_ or CO gas as the carbon source (Banerjee et al. 2014; Hoffmeister et al. 2016; Zhou et al. 2012) by mesophilic organisms. CO_2_ fixation by mixotrophy improves conversion of organic compounds to acetone, as shown by an engineered strain of *C. ljungdahlii* (Jones et al. 2016).

Among the various bioconversion applications, gas fermentation utilizing anaerobic acetogenic bacteria (acetogen) is attracting increasing attention. Gas fermentation is economically and environmentally friendly because inexpensive gaseous waste feedstock, such as steel mill waste gas or syngas primarily comprising CO and H_2_, can be used (Claassens et al. 2016; Durre and Eikmanns 2015; Liew et al. 2016). Whereas practical applications still demand higher productivity and cost-effective processes, the combination of bioreactive distillation as the purification process with gas fermentation can reduce waste and cost, in addition to engineering the metabolism of acetogens. The economic feasibility of acetone production from syngas by bioreactive distillation has been evaluated using hypothetical systems with engineered thermophilic strains of *Moorella thermoacetica* (Redl et al. 2017). The bioreactive distillation also has advantages to remove acetone that has inhibitory effect on the cell growth and could maintain high cell density without need to replace culture medium if appropriate bioreactors are used. However, the detailed metabolic design and construction of the thermophilic strains for gas fermentation, their availability, and, therefore, experimental data to support the system are missing.

This study genetically engineered the thermophilic homoacetogen *M. thermoacetica* to produce acetone from gaseous substrates at high temperature. We also developed a strategy to increase the carbon flux to acetone by genetic engineering and evaluated the productivity from CO_2_–H_2_, CO and CO–H_2_ as a model syngas. To our knowledge, this is the first study to provide strains for thermophilic gas fermentation of acetone.

## Materials and methods

### Bacterial strains and growth conditions

We used *M. thermoacetica* ATCC 39073 and its derivatives in this study (Table 1). Modified ATCC1754 PETC medium comprising 1.0 g of NH_4_Cl, 0.1 g of KCl, 0.2 g of MgSO_4_·7H_2_O, 0.8 g of NaCl, 0.1 g of KH_2_PO_4_, 0.02 g of CaCl_2_·2H_2_O, 2.0 g of NaHCO_3_, 10 mL of trace elements, 10 mL of Wolfe’s vitamin solution (Tanner 1989), and 1.0 mg of resazurin/L of deionized water was used as the basal medium (Tanner et al. 1993). The pH was adjusted to 6.9. The medium was prepared anaerobically by boiling and cooling under a N_2_–CO_2_ (80:20) mixed-gas atmosphere. After cooling, the medium was dispensed to 125-mL serum bottles under a N_2_–CO_2_ mixed-gas atmosphere. The serum bottles were crimp-sealed and autoclaved.

**Table 1:** Strains and plasmids used in this study

Strain or plasmid	Description	Source or reference
Strains		
*Escherichia coli*		
HST08	Cloning host	TaKaRa
TOP10	Modification host	Invitrogen
*M. thermoacetica*		
ATCC 39073	Wild-type strain	ATCC
Δ*pyrF*	*pyrF* gene was deleted	Kita et al. (2013)
pyrF::acetone	The thermophilic acetone operon was introduced into the *pyrF* region by using pHM17	This study
pduL2::acetone	The thermophilic acetone operon was introduced into the *pduL2* region by using pHM5. As a result, *pduL2* was knocked out	This study
Plasmids		
pBAD33	Backbone plasmid for methylation plasmids, Cm^r^	Guzman et al. (1995)
pBAD-M1281	pBAD33 carrying the Moth_1671, Moth_1672, and Moth_2281 for DNA methylation	Kita et al. (2013)
pK18mob	Backbone plasmid for transformation plasmids, Km^r^	Schäfer et al. (1994)
pK18-ldh	A transformation plasmid to introduce *ldh* into the *pyrF* region by using *pyrF* as a selection marker	Kita et al. (2013)
pK18-Δ*pduL2*::*ldh*	A transformation plasmid to introduce *ldh* into the *pduL2* region by using *pyrF* as a selection marker	Iwasaki et al. (2017)
pHM17	A transformation plasmid to introduce the thermophilic acetone operon into the *pyrF* region by using *pyrF* as a selection marker	This study
pHM5	A transformation plasmid to introduce the thermophilic acetone operon into the *pduL2* region by using *pyrF* as a selection marker	This study

Before starting culture, we added yeast extract and l-cysteine·HCl·H_2_O to reach a final concentration of 1.0 and 1.2 g/L, respectively. 2.0 g/L of fructose was added for routine cultivation and to examine acetone production from sugar. The final volume was adjusted to 50 mL. To add gas substrates, we replaced the headspace of the serum bottles by CO_2_–H_2_ (20:80) (0.1 MPa), or we added CO (0.04 MPa) and additional H_2_ (0.04 MPa) after replacing the headspace of the serum bottles with N_2_ gas at atmospheric pressure. The temperature was maintained at 55℃ with shaking at 180 rpm.

### Plasmid construction

We constructed two plasmids, pHM17 and pHM5, to introduce the thermophilic acetone operon into the *pyrF* or the *pduL2* region of the chromosome in *M. thermoacetica* (Table 1). We synthesized the thermophilic acetone operon under the constitutive glyceraldehyde-3-phosphate dehydrogenase (G3PD) promoter after codon optimization of the four genes encoding acetone biosynthetic enzymes for expression in *M. thermoacetica* (GenScript). The genes constituting the thermophilic acetone operon were *ctfA* (Tmel_1136) and *ctfB* (Tmel_1135) from *Thermosipho melanesiensis*, *thl* (TTE0549) from *Caldanaerobacter subterraneus* subsp.* tengcongensis*, and *adc* (CA_P0165) from *C. acetobutylicum*. The open reading frames coding these four genes were driven by the constitutive G3PD promoter (Kita et al. 2013), and the gene order was determined on the basis of the biochemical information about the enzymes: activity, stability, and complex formation (Zeldes et al. 2018). Each gene was separated by an intergeneic spacer with a ribosome-binding site, and the DNA fragment synthesized was amplified by polymerase chain reaction (PCR) using KOD plus ver.2 (Toyobo Co., Ltd., Osaka, Japan) and this synthetic operon was inserted into the plasmids with a *pyrF* marker in either the *pyrF* or the *pduL2* region using the In-Fusion HD cloning kit (Clontech Laboratories, TaKaRa Bio, Shiga, Japan). We used pK18-ldh (Kita et al. 2013) or pK18-Δ*pduL2*::*ldh* (Iwasaki et al. 2017) as a template to amplify the plasmids (Table 1). Table 2 lists the primers used for PCR. We used JK50 and JK51 to amplify the insert and JK52 and JK53 to amplify plasmid backbones. Finally, we cloned the constructed plasmids in *E. coli* HST08 and confirmed the DNA sequences using Sanger sequencing.

**Table 2:** PCR primers used in this study

Name	Sequence (5′ to 3′)
JK50	GGTGAAATAATAACTGGACGGTTGCCAAGTACCG
JK51	ATGAAAGCAGGCCGATTACTTCAGATAATCGTAGATCACTTCGG
JK52	TCGGCCTGCTTTCATGCTTG
JK53	AGTTATTATTTCACCATCTCTATTTCCGCC
JK226	GGCCGCCGCCATTTAGCATATCAAGAG
JK227	GCCGCAAATGCTGGTAAAGGCTATC
1181-up-F	CGTTCAATAGGAAGACCACAG
1181-dw-R	GCAGTAAGCTGTATCGCAATG

### Transformation and selection of mutants

We performed the genetic transformation of *M. thermoacetica*, as previously described (Kita et al. 2013). All procedures were performed under aerobic conditions, except for cell growth. Briefly, we cultured the *M. thermoacetica* Δ*pyrF* mutant to the mid-log phase in basal medium supplemented with 2 g/L of fructose as a carbon source and 10 µg/mL of uracil instead of yeast extract, and harvested it by centrifugation. Next, the cells were washed twice with 272 mM sucrose solution and used for electroporation with methylated DNA in the *E. coli* TOP10-harboring plasmid pBAD-M1281. The transformed cells were then cultured at 55 °C for 24–48 h with a low uracil concentration of 1 µg/mL before inoculation to the modified ATCC1754 PETC medium containing agar without uracil or yeast extract in roll tubes (Hungate 1969). The roll tubes were cultured at 55 °C, and the colonies were subcultured to confirm the insertion of the thermophilic acetone operon by using PCR. We used the primer set JK226 and JK227 to amplify the *pyrF* region and 1181-up-F and 1181-up-R to amplify the *pduL2* region. The constructed strain with higher acetone productivity has been deposited to NITE (NITE AP-03217).

### Analytical methods

We sampled and analyzed 1 mL of the culture medium at each time point and calculated the dry cell weight using the optical density (OD) at 600 nm (1 g [dry cell weight]/L = 0.383 OD) (Iwasaki et al. 2017). The culture supernatant was analyzed for the amount of fructose, formate, acetate, and acetone using high-performance liquid chromatography (HPLC) (LC-2000 Plus HPLC; Jasco, Tokyo, Japan) equipped with a refractive index detector (RI-2031 Plus; Jasco), a Shodex RSpak KC-811 column (Showa Denko, Kanagawa, Japan), and a Shodex RSpak KC-G guard column (Showa Denko) at 60 °C. Ultrapure water containing 0.1% (v/v) phosphoric acid was used as the mobile phase at a flow rate of 0.7 mL/min, and crotonate was used as an internal standard (Miura et al. 2014). The gas composition in the headspace of the serum bottles was analyzed by using GC-8A gas chromatography (Shimadzu, Kyoto, Japan) equipped with a thermal conductivity detector and a stainless steel column packed with activated carbon at 70 °C. Argon was used as the carrier gas (Miura et al. 2014). The amount of dissolved carbonate in the culture medium was measured by using a total organic carbon analyzer (TOC-L; Shimadzu).

### Nucleic acid sequences

The nucleic acid sequences of the synthetic acetone operon have been deposited to GenBank (accession number MW436696).

## Results

### Design and construction of genetically engineered M. thermoacetica strains for thermophilic acetone production

**Fig. 1:**
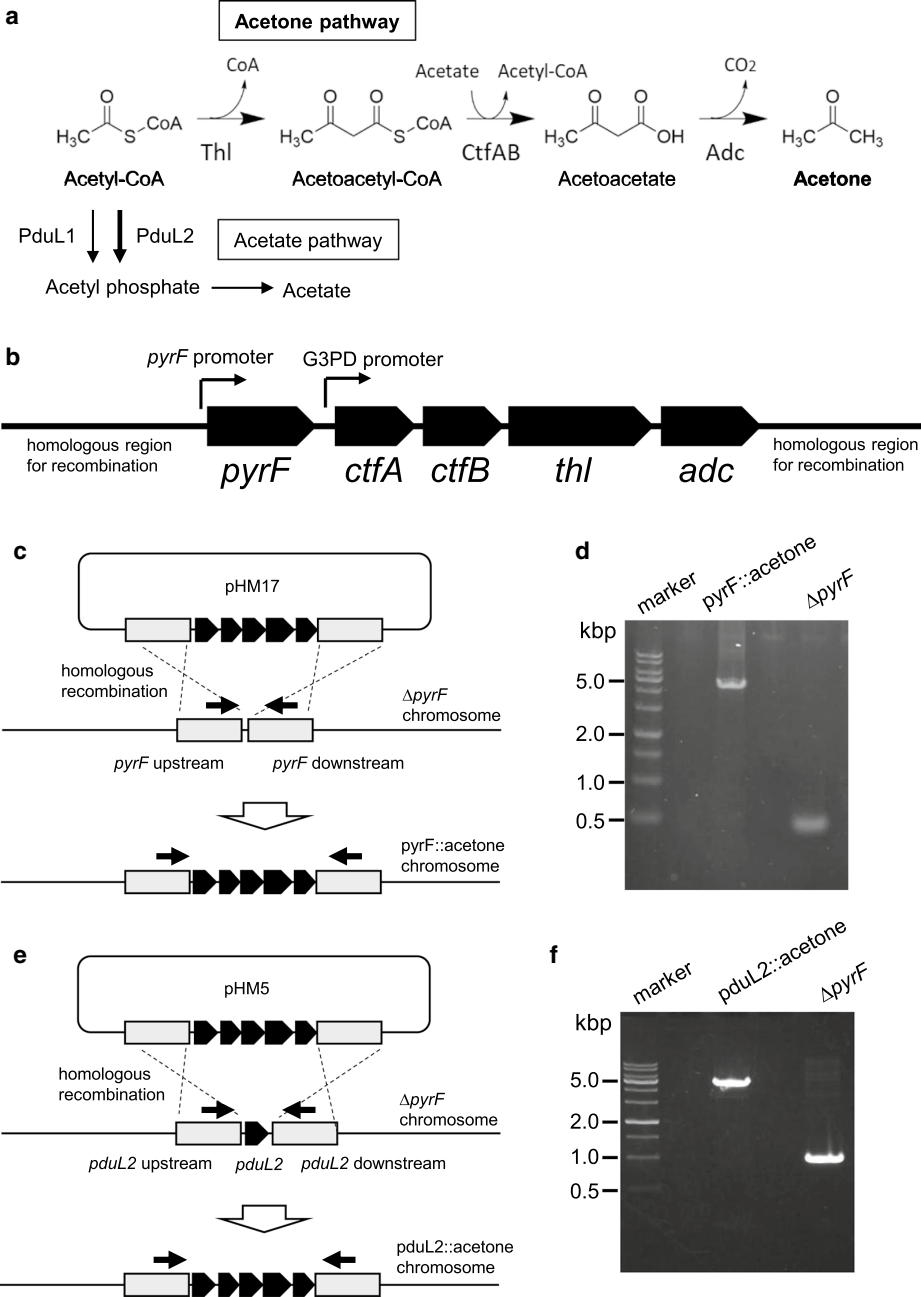
Design and construction of acetone-producing *Moorella thermoacetica* strains.** a** Acetone production pathway. Two molecules of Ac-CoA are converted to one molecule of acetone via three reactions using one molecule of acetate. The reactions release a CoA molecule, an Ac-CoA molecule, and a CO_2_ molecule, in addition to an acetone molecule. Acetate pathway from Ac-CoA is also shown. Ac-CoA is converted to acetyl phosphate by phosphotransacetylase that is encoded by *pduL1* as well as *pduL2*, followed by conversion to acetate. **b** Schematic representation of the synthetic acetone production operon. Genes and promoters are shown by block and fine arrows, respectively. **c**, **e** Schematic representations of the introduction of the synthetic thermophilic acetone operon by homologous recombination into the *pyrF* (**c**) and the *pduL2* (**e**) region. The gray boxes highlight DNA regions used for recombination, and the line arrows represent primers used for PCR. The primer set to amplify the *pyrF* region is JK226 and JK227 and that for the *pduL2* region is 1181-up-F and 1181-dw-R. **d**, **f** Verification of the presence of the thermophilic acetone operon in the *pyrF* (**d**) and the *pduL2* (**f**) region. The genomic region of each gene was amplified by PCR, and the size shift due to the insertion was confirmed. The size of the PCR product of the *pyrF* region shifted from 0.5 to 4.8 kb by introducing the thermophilic acetone operon and the selection marker (**d**). Similarly, the PCR product of the *pduL2* region shifted from 1.0 to 4.9 kb (**f**)

**Fig. 2:**
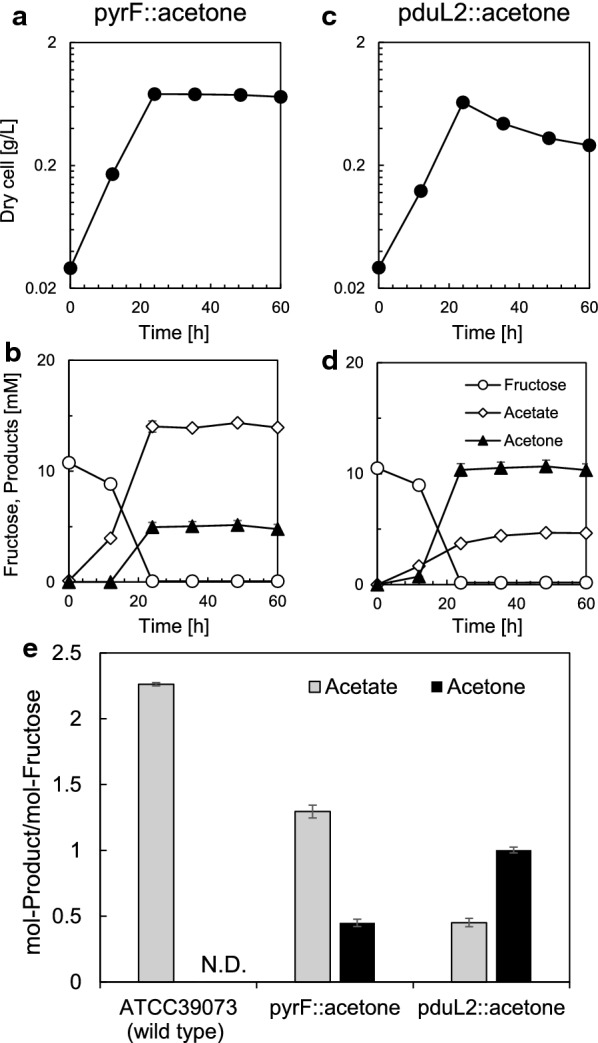
Acetone and acetate production from fructose by the recombinant *Moorella thermoacetica* strains.** a**, **c** Dry cell weight according to the OD for the pyrF::acetone strain (**a**) and pduL2::acetone strain (**c**). **b**, **d** Concentration of fructose and excreted metabolites in the culture supernatant measured by HPLC for the pyrF::acetone strain (**b**) and the pduL2::acetone strain (**d**). Data represent the mean with SDs of three biological replicates. Most error bars are smaller than the symbols of data plots. **e** The acetone and acetate productivity per 1 mol of fructose is shown with black (acetone) and gray (acetate) bars. The productivity was calculated based on the measurement after complete consumption of the supplemented fructose. The parental strain, ATCC 39073 (wild type), which does not produce acetone is shown for comparison. Data represent the mean with SDs of three biological replicates

*Moorella thermoacetica* grows at 45 °C–65 °C. A pathway for thermophilic acetone production, which functions up to 70 °C, has been proposed with enzyme candidates (Zeldes et al. 2018). This pathway converted two molecules of acetyl-CoA (Ac-CoA) to acetoacetyl-CoA (Acac-CoA) as the start reaction by thiolase (Thl), followed by two reactions that produced acetoacetate (Acac) and acetone (Fig. 1a). When Acac was produced by CoA transferase (CtfAB), one molecule of acetate was required to receive a CoA molecule from Acac-CoA. *M. thermoacetica* provides both Ac-CoA and acetate that are used as substrates in this pathway on sugars or gaseous substrates. In *M. thermoacetica*, Ac-CoA is an intermediate to produce acetate as the end metabolite. We selected thermophilic enzymes and designed the acetone biosynthesis operon (Fig. 1b), and the synthetic thermophilic acetone operon was successfully introduced into the wild-type (WT) background of *M. thermoacetica* (Fig. 1c and d). Next, we cultured the pyrF::acetone strain in basal medium supplemented with fructose at 55 °C (optimum growth temperature). Acetone was successfully produced and released into the culture supernatant, indicating functional expression of the enzymes. Consistent with the absence of homologous genes encoding secondary alcohol dehydrogenase in the genome of *M. thermoacetica*, the produced acetone was not converted to isopropanol unlike the case of some acetogens (Hoffmeister et al. 2016; Kopke et al. 2014; Pierce et al. 2008). However, we detected a large amount of acetate (about three times more than acetone) in the culture supernatant, indicating that Ac-CoA is mostly converted to acetate (Fig. 2a, b and e).

### Deletion of pduL2 and preservation of pduL1 lead to higher acetone production

The introduction of the thermophilic acetone operon did not cause high acetone production by *M. thermoacetica*. We hypothesized that the Thl responsible for the first reaction could not capture Ac-CoA because of the abundant phosphotransacetylase activity by PduL1 and PduL2 in *M. thermoacetica*. PduL2 showed more than a tenfold lower Michaelis constant (*K*_m_ = 0.04 mM) against Ac-CoA compared to PduL1 (*K*_m_ = 0.49 mM), while Thl showed a *K*_m_ value of 0.27 mM (Breitkopf et al. 2016; Loder et al. 2015). Although we did not measure PduL1, PduL2, Thl, and Ac-CoA levels in the cells, the low *K*_m_ value of PduL2 might explain the abundant acetate production in the pyrF::acetone strain. To test this hypothesis, we knocked out *pduL2* and measured the acetone production. We introduced the thermophilic acetone operon to replace the *pduL2* coding region, which enabled us to delete *pduL2* and introduce acetone biosynthetic genes at the same time (Fig. 1e and f). We cultured the pduL2::acetone strain in basal medium with fructose and found a significant increase in acetone production and decrease in acetate production, resulting in 1.0 ± 0.02 mol-acetone/mol-fructose and 0.45 ± 0.03 mol-acetate/mol-fructose (Fig. 2c–e). The acetone–acetate ratio was 0.35 ± 0.03 in the case of the pyrF::acetone strain, but increased to 2.23 ± 0.21 in the pduL2::acetone strain. Acetone production was dominant over acetate production, and thus, we successfully directed more Ac-CoA pool to the acetone pathway.

### Thermophilic acetone production from CO_2_–H_2_

**Fig. 3:**
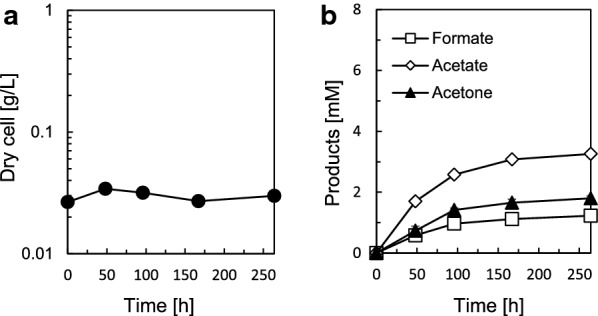
Growth and metabolite profile of the pduL2::acetone strain in CO_2_–H_2_ as the substrate. **a** Dry cell weight according to the OD. **b** Concentration of the excreted metabolites in the culture supernatant measured by HPLC. Data represent the mean with SDs of three biological replicates. Most error bars are smaller than the symbols of data plots

We aimed to produce acetone from gaseous substrates with high productivity by using the pduL2::acetone strain. CO_2_ and H_2_ are the best-studied form of substrates for autotrophic acetogenesis. First, we tested CO_2_ as a carbon source and H_2_ as an energy source. To set up the culture, the bacterial strain was grown in basal medium supplemented with fructose, and we used this culture to inoculate fresh medium with CO_2_–H_2_ in the headspace of the vial for the pre-culture. This step was performed for bacterial cells to completely consume fructose, followed by adaptation to CO_2_–H_2_ metabolism. We inoculated fresh medium supplemented with CO_2_–H_2_ by using the adapted cells and recorded the culture profile. There was almost no growth during 254 h of cultivation time (Fig. 3a). Excreted metabolites accumulated over time (Fig. 3b), indicating that the cells were metabolically active. Acetone was successfully produced in CO_2_–H_2_, reaching 1.8 ± 0.08 mM in the culture supernatant after 254 h. Acetate production reached 3.3 ± 0.09 mM, which was dominant over acetone production, although the pduL2::acetone strain was engineered to have a higher carbon flux to acetone in the culture supplemented with fructose. In addition, formate, which is an intermediate in the Wood–Ljungdahl pathway (WLP), also accumulated in the culture supernatant, reaching 1.2 ± 0.12 mM, indicating that the metabolic flow of the WLP is affected.

### Thermophilic acetone production from CO or syngas

**Fig. 4:**
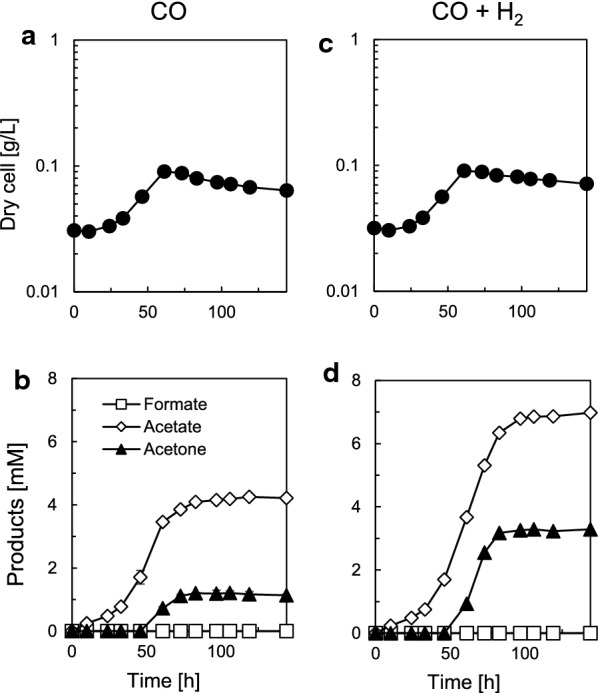
Growth and metabolite profile of the pduL2::acetone strain in CO and CO–H_2_. **a**, **c** Dry cell weight according to the OD. **b**, **d** Excreted metabolites measured by HPLC. Profiles of CO (**a**, **b**) and CO–H_2_ (**c**, **d**) are shown, respectively. The values shown are means of three biological replicates, and error bars represent the SD. Some error bars showing a small error range overlapped with symbols of data plots

The pduL2::acetone strain showed no growth in CO_2_–H_2_, so we tested a more energetically favorable gas, CO, for acetone production with autotrophic growth. *M. thermoacetica* uses CO as the energy source and shows a higher biomass than in H_2_ because of higher adenosine triphosphate (ATP) generation (Hu et al. 2016; Kerby and Zeikus 1983). To initiate the culture, we adapted the pduL2::acetone strain to CO in the same way as CO_2_–H_2_, especially because CO inhibits *M. thermoacetica* growth without adaptation (Kerby and Zeikus 1983). The bacterial cells proliferated using CO as a carbon and energy source, in contrast to CO_2_–H_2_ as observed by an obvious increase of the cellular biomass (Fig. 4a). We also observed acetone and acetate production, and their maximum concentration was 1.1 ± 0.04 and 4.2 ± 0.08 mM, respectively (Fig. 4b). The acetone–acetate ratio was 0.27 ± 0.01, which was again acetate dominant. No formate production was observed in contrast to CO_2_–H_2_, indicating that the metabolic flow of WLP was not affected. Thus, the pduL2::acetone strain autotrophically grew and produced acetone in CO.

Syngas mainly comprises CO and H_2_ and applicable substrates for sustainable gas fermentation. We tested a 1:1 CO and H_2_ mixture as a model case. The culture was set up in the same way as in CO. Whereas the culture profile showed almost the same biomass and growth rate as in CO without H_2_ (Fig. 4c), the acetone productivity significantly improved from 1.1 ± 0.04 to 3.3 ± 0.10 mM (Fig. 4b and d) from the same amount of CO, and the increment was higher compared to acetate (from 4.2 ± 0.08 to only 7.0 ± 0.10 mM), indicating enhanced carbon flux to the acetone production pathway. The acetone–acetate ratio increased from 0.27 ± 0.01 to 0.47 ± 0.01 and was still acetate dominant but was higher compared to with CO. In addition, there was no formate accumulation. Therefore, by adding H_2_, the acetone productivity from the same amount of carbon source increased, and the maximum specific acetone production rate also increased from 0.04 ± 0.003 to 0.09 ± 0.005 g-acetone/g-dry cell/h.

The growth and metabolite profiles of the autotrophic acetone production in CO-containing gases can be compared by the fermentation parameters summarized in Table 3. The electrons derived from H_2_ seemed invested to acetone and acetate production rather than cellular biomass, because H_2_ supplementation did not affect cell growth. It was also indicated that the electron input was directed to acetone rather than acetate.

**Table 3:** Fermentation parameters for acetone production from CO-containing gases

Substrate	C_H_2_/s^a^ (mol/mol)^b^	Yield for substrates					Recovery		Specific growth rate (h^−1^)	Specific production rate of acetone (g-acetone/g-cell/h)
		Y_biomass/s (g/mol)	Y_acetate/s (mol/mol)	Y_acetone/s (mol/mol)	Y_CO_2_/s (mol/mol)	Y_H_2_/s (mol/mol)	Carbon (%)	Electron (%)		
CO	NA ^c^	2.04 ± 0.03	0.16 ± 0.00	0.04 ± 0.00	0.49 ± 0.02	0.01 ± 0.00	101 ± 1	115 ± 2	0.03 ± 0.00	0.04 ± 0.00
CO–H_2_	0.48 ± 0.02	1.04 ± 0.04	0.13 ± 0.01	0.06 ± 0.00	0.13 ± 0.02	ND ^d^	120 ± 2	110 ± 2	0.03 ± 0.00	0.09 ± 0.00

^a^ s = CO + H_2_

^b^ The molar ratio of H_2_ in consumed energy gas (CO and H_2_)

^c^Not applied

^d^Not determined

## Discussion

Acetone production by introduction of a thermophilic acetone biosynthetic operon in *M. thermoacetica* showed that the selected proteins were functionally expressed. However, when WT background was used as the host, the end product was acetate dominant. To increase the acetone productivity over acetate, we made use of a unique feature of *M. thermoacetica* that two functional phosphotransacetylase genes (*pduL1* and *pduL2*) are involved in acetate production. In the pyrF::acetone strain, three enzymes, PduL1 and PduL2 for acetate production and Thl for acetone production, compete to process Ac-CoA (Fig. 1a). PduL2 shows a lower *K*_m_ against Ac-CoA compared to PduL1 and Thl, and was likely to cause dominant acetate production. The removal of *pduL2* successfully enhanced acetone production in the pduL2::acetone strain. As a result, the acetone–acetate ratio significantly increased to be comparable to the engineered *C. ljungdahlii* using lactose-inducible promoter for expression of the acetone synthesis enzymes under fructose or CO fermentation growth conditions (Banerjee et al. 2014). The acetone production ratio of our strain from CO was further increased by adding H_2_ as discussed below. Previously, the effect of *pduL2* knockout was also seen in our report that partial disruption of the acetate production pathway by *pduL2* knockout enhanced lactate production in the metabolically engineered strains to produce lactate (Iwasaki et al. 2017). The production of lactate, which is provided by a reduction of pyruvate, was significantly enhanced by eliminating *pduL2* because of the increased available Ac-CoA pool and therefore pyruvate, while *pduL1* disruption had a marginal effect.

It is also useful to control the metabolic flow toward acetate by eliminating only *pduL2* to maintain the autotrophy on syngas. Acetate not only acts as a substrate for acetone synthesis but also sustains sufficient net ATP production by substrate-level phosphorylation. The pduL2::acetone strain maintains autotrophy in CO-containing gases, while autotrophic growth collapses in CO_2_–H_2_.

The autotrophy of acetogens is energetically at the limit of thermodynamics (Schuchmann and Muller 2014). When *M. thermoacetica* grows in CO_2_–H_2_, the net ATP production would be calculated to be only 0.5 mol-ATP/mol-acetate (Schuchmann and Muller 2014, and see also Online Resource Additional file 1: Figure S1):

$${\text{2CO}}_{{2}} + {\text{ 4H}}_{{2}} \to {\text{ Acetate }} + {\text{ 2H}}_{{2}} {\text{O }}\left( { + 0.{\text{5 ATP}}} \right)$$

The positive ATP level is possible only when acetate is produced, because the ATP level is –0.5 at the point of Ac-CoA production (–0.5 mol-ATP/mol-Ac-CoA). The consumed ATP is complemented by acetate production, which yields 1 mol-ATP/mol-acetate. Because acetone production uses both Ac-CoA and acetate as substrates, diverging Ac-CoA to the acetone pathway lowers ATP production derived from substrate-level phosphorylation. When acetone is produced at the maximum efficiency from H_2_ and CO_2_, incorporating all the produced acetate, the net ATP is zero (Additional file 1: Fig. S1):

$${\text{3CO}}_{{2}} + {\text{ 8H}}_{{2}} \to {\text{ Acetone }} + {\text{ 5H}}_{{2}} {\text{O }}\left( { + 0{\text{ ATP}}} \right)$$

The pduL2::acetone strain did not grow in CO_2_–H_2_ (Fig. 3a), which can be explained by the low net ATP production. In fact, formate accumulation was observed in the metabolite analysis (Fig. 3b), indicating ATP shortage. In the WLP, formate is produced by the reduced nicotinamide adenine dinucleotide phosphate (NADPH)-dependent reduction of CO_2_ in *M. thermoacetica*. This formate is then ligated to tetrahydrofolate (THF) via an ATP-dependent reaction (Schuchmann and Muller 2014). Therefore, low net ATP production causes ATP shortage and formate accumulation. In addition to formate, acetone and acetate are produced by the pduL2::acetone strain, indicating that the cells were metabolically active but not able to grow. Acetate production is linked to ATP production by substrate-level phosphorylation, and the ATP produced is used for cellular maintenance and formate ligation to THF. In the case of the engineered *A. woodii* producing acetone from CO_2_–H_2_, highly abundant acetate compared to acetone was produced to provide sufficient ATP production and maintain its autotrophic growth (Hoffmeister et al. 2016). ATP shortage is a challenge for the autotrophic acetone production with low level of acetate as the byproduct in CO_2_–H_2_.

In contrast, when a CO-containing gas is used, acetone production occurs as follows (Additional file 1: Fig. S1):

$${\text{8CO }} + {\text{ 3H}}_{{2}} {\text{O }} \to {\text{ Acetone }} + {\text{ 5CO}}_{{2}} \left( { + {\text{2 ATP}}} \right)$$

In addition, when H_2_ is supplied (Additional file 1: Fig. S1),

$${\text{3CO }} + {\text{ 5H}}_{{2}} \to {\text{ Acetone }} + {\text{ 2H}}_{{2}} {\text{O }}\left( { + 0.{\text{75 ATP}}} \right).$$

In both cases, the net ATP is positive to sustain autotrophic growth. It has been discussed that when acetate is not formed from Ac-CoA to divert metabolic pathway, the WLP would be severely ATP limited (Fast and Papoutsakis 2012). However, the acetone pathway utilizes acetate that is formed from Ac-CoA (Fig. 1a), which is advantageous to supply ATP. Applying H_2_ enhances the acetone production per consumed CO from 0.13 mol-acetone/mol-CO without H_2_ to 0.33 mol-acetone/mol-CO with H_2_. In theory, acetone production should be 2.5 times higher with H_2_ supplementation, leaving no acetate as a by-product, when the reaction proceeds at the maximum efficiency. Our experiment with the pduL2::acetone strain showed that H_2_ supplementation significantly improved acetone production to ~ 2.5 times higher (Fig. 4) compared to only CO supplementation, although the amount of acetate also increased to ~ 1.7 times higher. The remaining acetate not incorporated into the acetone pathway indicates that acetone productivity could be potentially improved in both CO and CO–H_2_ by tuning the final amount of acetate to zero without losing autotrophic growth.

One explanation for the abundant acetate that remained in our gas fermentation is due to the limit of enzymatic reactions, such as the CoA transferase that transfers CoA from Acac-CoA to acetate. An increase in acetate concentration is required to start solventogenesis in *C. acetobutylicum*, because CoA transferase shows a high *K*_m_ of 1200 mM against acetate, while it shows a low *K*_m_ of ~ 7–56 µM against Acac-CoA (Wiesenborn et al. 1989). Although we did not analyze the enzymatic properties of CoA transferase from *T. melanesiensis*, it is conceivable that the enzyme has a high *K*_m_ against acetate and that the acetate concentration is a limiting factor. In fact, culture on fructose provided much higher concentration of acetate (Fig. 2d). Further examination and optimization of the selected enzymes would contribute to higher productivity, in addition to the experiments such as utilization of bioreactors to provide abundant substrates to reach high concentrations of the products including acetate. It is also possible that PduL1, which is responsible for remained production of acetate in the pduL2::acetone strain, was expressed higher on the gaseous substrates. This is because when the acetone production rate was compared between fructose culture and CO–H_2_ culture, both showed similar rates (0.12 ± 0.01 g-acetone/g-dry cell/h on fructose and 0.09 ± 0.00 g-acetone/g-dry cell/h on CO–H_2_, respectively, calculated from Figs. 2d and 4d). In other words, ATP would not be limiting factor in CO–H_2_ culture, owing to the sufficient production of acetate linked to ATP production. This level of acetate might be necessary for the autotrophic acetone production at this rate. Otherwise, repression of PduL1 expression or replacement of the enzyme itself with its homologue with larger *K*_m_ value would be able to reduce acetate production and increase acetone productivity.

Finally, yet importantly, acetone production by engineering acetogenic metabolism has the benefit of redox balance, in addition to the use of Ac-CoA and acetate as substrates. In many cases of redox balance by native and engineered metabolism, unused electrons in the metabolic pathways are dedicated (or disposed of) to the reactions for end products. The redox imbalance is often a cause of low yield of end products and poor bacterial growth. The acetone pathway from Ac-CoA requires no reducing energy, so the redox balance in acetone production is difficult by using, for example, an *E. coli* system under anaerobic conditions because of the absence of reactions for unused electrons (Bermejo et al. 1998). However, the WLP produces acetate as the sole end product via Ac-CoA with redox balance, requiring no redox reactions from Ac-CoA through acetate. Therefore, it is beneficial to use the WLP to produce acetone with regard to the redox balance as well.

In this report, we successfully engineered a thermophilic acetogen *M. thermoacetica* for autotrophic acetone production from syngas. Acetone productivity improves by partial deletion of the production pathway for acetate used as a substrate as well as for energy conservation. *M. thermoacetica* grows at a temperature higher than the boiling point of acetone (58 °C); therefore, thermophilic processes of gas fermentation producing volatile chemicals could be built and evaluated. Although further study would be needed to improve the productivity for realization of the industrial applications, the gas fermentation process can be simpler and more cost-effective than before by incorporating a purification process by distillation of the acetone produced from gaseous substrates.

## Supplementary Information

**Additional file 1: Figure S1. **Schematic representation of energy conservation in acetone-producing *Moorella thermoacetica*.

## Data Availability

All data collected or analyzed during this study are included in this published article.
