# Supplementary material for: Metabolic engineering of Moorella thermoacetica for thermophilic bioconversion of gaseous substrates to a volatile chemical
Source: AMB Express. 2021 Apr 23;11:59. doi: 10.1186/s13568-021-01220-w (PMC8065083; doi:10.1186/s13568-021-01220-w)
Supplement: Supplementary file 1 — Additional file 1: Figure S1. Schematic representation of energy conservation in acetone-producing Moorella thermoacetica. [file 13568_2021_1220_MOESM1_ESM.docx]

***^AMB Express^***

**Supplementary Information for:**

**Metabolic engineering of *Moorella thermoacetica* for thermophilic bioconversion of gaseous substrates to a volatile chemical**

Junya Kato^1,3^, Kaisei Takemura^1,3^, Setsu Kato^1^, Tatsuya Fujii^2^, Keisuke Wada^2^, Yuki Iwasaki^2^, Yoshiteru Aoi,^1^ Akinori Matsushika^1,2^, Katsuji Murakami^2^ and Yutaka Nakashimada^1, *^

^*^Corresponding author at: E-mail [nyutaka@hiroshima-u.ac.jp](mailto:nyutaka@hiroshima-u.ac.jp), Phone No.: +81-82-424-4443

^1^Graduate School of Integrated Sciences for Life, Hiroshima University, 1-3-1 Kagamiyama, Higashihiroshima, Hiroshima, 739-8530, Japan

^2^National Institute of Advanced Industrial Science and Technology (AIST), 3-11-32 Kagamiyama, Higashihiroshima, Hiroshima, 739-0046, Japan

^3^J.K. and K.T. contributed equally to this work.

**^This Supplementary Information file contains Fig. S1.^**

**
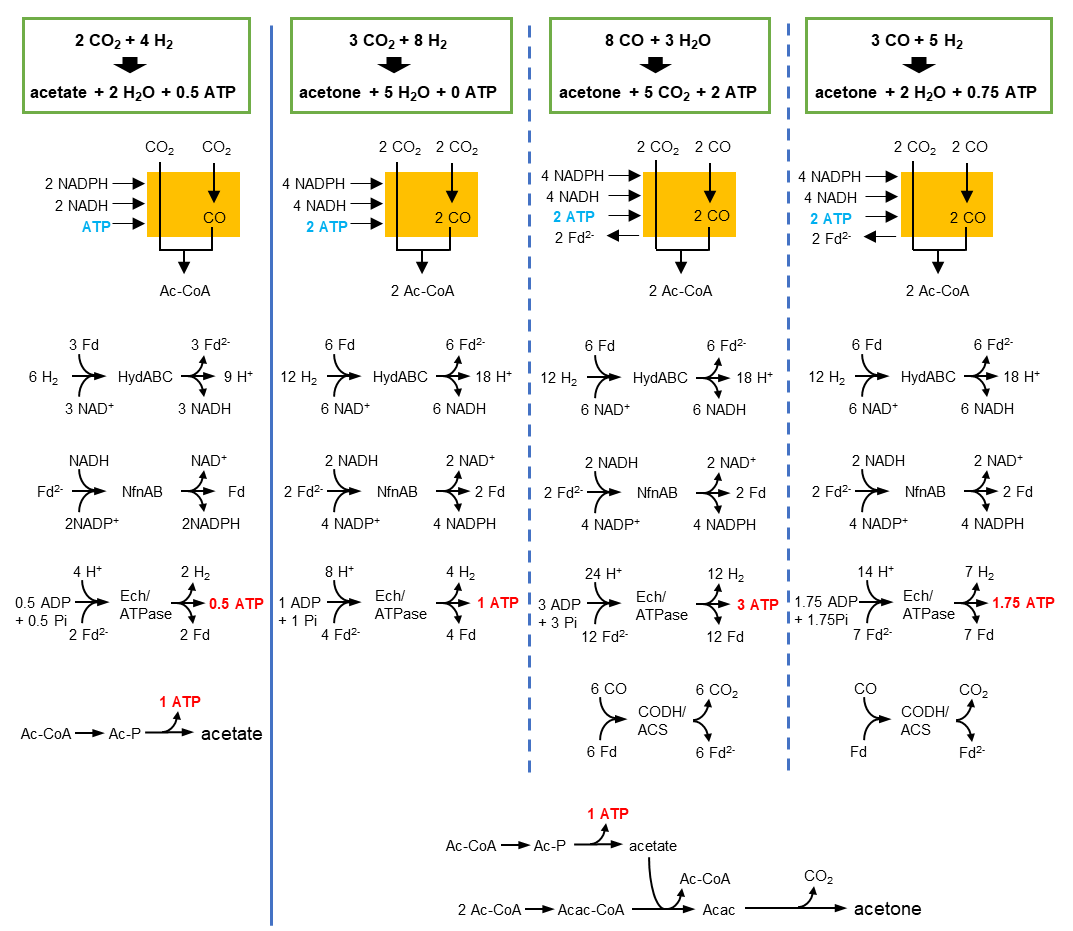
**

**Supplementary Information** **Figure S1. Schematic representation of energy conservation in acetone-producing *Moorella thermoacetica*.** ATP generation for acetate or acetone production as the sole end product is shown. The estimation and calculation were according to Schuchmann and Muller (2014) and a part of the figure was adopted from Redl et al. (2017). The orange box is a part of the WLP to convert CO_2_ to Ac-CoA. HydABC and NfnAB are protein complexes to bifurcate electrons. Acac, acetoacetate; Acac-CoA, acetoacetyl–coenzyme A; Ac-P, acetyl phosphate; Ac-CoA, acetyl–coenzyme A; CODH/ACS, CO dehydrogenase/acetyl–coenzyme A synthase; Ech/ATPase, Ech complex/F_0_F_1_ adenosine triphosphate synthase; Fd, ferredoxin; ATP, adenosine triphosphate.
